# Genomics based analysis of interactions between developing B-lymphocytes and stromal cells reveal complex interactions and two-way communication

**DOI:** 10.1186/1471-2164-11-108

**Published:** 2010-02-12

**Authors:** Jenny Zetterblad, Hong Qian, Sasan Zandi, Robert Månsson, Anna Lagergren, Frida Hansson, David Bryder, Nils Paulsson, Mikael Sigvardsson

**Affiliations:** 1 Department for Hematopoietic Stemcell Biology, Lund Stem Cell Center, BMC B12, 221 84 Lund, Sweden; 2 Institution for Clinical and Experimental Science, Linköping Universitet, 581 85 Linköping, Sweden; 3 Department for Immunology, Lund University, BMC D14, 221 84 Lund, Sweden

## Abstract

**Background:**

The use of functional genomics has largely increased our understanding of cell biology and promises to help the development of systems biology needed to understand the complex order of events that regulates cellular differentiation *in vivo*. One model system clearly dependent on the integration of extra and intra cellular signals is the development of B-lymphocytes from hematopoietic stem cells in the bone marrow. This developmental pathway involves several defined differentiation stages associated with specific expression of genes including surface markers that can be used for the prospective isolation of the progenitor cells directly from the bone marrow to allow for *ex vivo *gene expression analysis. The developmental process can be simulated *in vitro *making it possible to dissect information about cell/cell communication as well as to address the relevance of communication pathways in a rather direct manner. Thus we believe that B-lymphocyte development represents a useful model system to take the first steps towards systems biology investigations in the bone marrow.

**Results:**

In order to identify extra cellular signals that promote B lymphocyte development we created a database with approximately 400 receptor ligand pairs and software matching gene expression data from two cell populations to obtain information about possible communication pathways. Using this database and gene expression data from NIH3T3 cells (unable to support B cell development), OP-9 cells (strongly supportive of B cell development), pro-B and pre-B cells as well as mature peripheral B-lineage cells, we were able to identify a set of potential stage and stromal cell restricted communication pathways. Functional analysis of some of these potential ways of communication allowed us to identify BMP-4 as a potent stimulator of B-cell development *in vitro*. Further, the analysis suggested that there existed possibilities for progenitor B cells to send signals to the stroma. The functional consequences of this were investigated by co-culture experiments revealing that the co-incubation of stromal cells with B cell progenitors altered both the morphology and the gene expression pattern in the stromal cells.

**Conclusions:**

We believe that this gene expression data analysis method allows for the identification of functionally relevant interactions and therefore could be applied to other data sets to unravel novel communication pathways.

## Background

The development of mature blood cells from haematopoietic stem cells is a process involving a gradual loss of multi-lineage potential and a subsequent gain of lineage restricted cellular features. The maturation process is reflected in surface marker expression allowing for sorting of cells at defined stages of development and for detailed functional and molecular analysis of stage specific events [1,2]. This has revealed that the differentiation process is critically dependent on a set of transcription factors that appears to act in a hierarchical and coordinated manner to activate the correct genes and allow the developmental pathway to proceed [3]. However, the action of transcription factors and the outcome of the developmental process are also highly dependent on communication with other cells in the bone marrow (BM) micro-environment [4,5]. One of the most carefully investigated developmental pathways in the BM is the differentiation of B-lymphoid cells. The earliest B cell progenitors are responsive to the stimulatory action of the chemokine CXCL12 (SDF-1) [6,7] produced by BM stromal cells [8], acting via the CXCR4 receptor on the pro-B cells [9,10]. CXCL12 is produced by stromal cells scattered in the BM, possibly creating a distinct anatomical niche for the earliest stages of B-lymphoid development [8]. These early cells are also supported by the action of FL-ligand, that via the FLT-3 receptor [11,12] stimulate lymphoid primed multipotent progenitors (LMPPs [13]) to proceed into the lymphoid lineages [14]. The subsequent developmental stage in B-lymphocyte development display a critical need for the cytokine IL-7 and mice deficient in either the cytokine or the α component (IL-7Rα) of the hetero-dimeric receptor, display disturbances in differentiation in both B and T lymphocyte development [15,16]. The phenotype observed in these mice is further enhanced by the combined disruption of both the IL-7Rα and FL genes where the block of B cell development is nearly complete [17,18]. It has been reported that ectopic expression of a second ligand for the IL7Rα subunit, Thymic Stromal Lympho Protein (TSLP) [19], can rescue the B-cell defects in IL-7 deficient mice arguing for partially redundant functions of IL-7 and TSLP [20]. However, mice deficient in TSLP develop an apparently normal B-cell compartment suggesting that the central factor *in vivo *is IL-7 [21]. Later stages of B cell development has been suggested to be dependent on contact with supporting cells since hematopoietic cells deficient in RANK ligand are impaired in their ability to reconstitute the B220^+^IgM^+ ^immature progenitor compartment upon transplantation into wild type mice [22]. Thus, it is apparent that normal B-lymphopoiesis is dependent on communication and contact between the developing cell and surrounding cells like BM stroma cells [5]. Even if a set of critical factors for normal development has been identified, it is reasonable to presume that the cells are exposed to a much larger number of signals that may influence normal as well as malignant B-cell growth and maturation.

In order to identify potential communication pathways involved in the regulation of blood cell development, we have developed a bioinformatic tool, Genomics based Cell-cell Interaction analysis (GCINT) where gene expression microarray data from two different cell types can be used to unravel possible means of signaling in the cellular interphase. The analysis is based on a database containing verified receptor ligand pairs and a second database that allows input of normalized gene expression data from two different cell types. The data is then analyzed so to unravel potential means of communication based on matching expression of receptors and ligands. This allowed us to identify novel potential communication pathways, some of which were functionally investigated by *in vitro *differentiation of hematopoietic progenitors. In addition, the analysis revealed that the presence of blood cells has a direct impact on the gene expression of the stromal cells suggesting that the interaction involve a complex two-way communication.

## Methods

### GCINT analysis

GCINT is a database made in Microsoft Access, specialized in matching known receptor-ligand interactions using the locus link number from the microarrays. All arrays used for GCINT analysis were normalized together using RMA Express adding absent/present classification. Known receptor-ligand pairs identified by NCBI http://www.ncbi.nlm.nih.gov and Genecards http://www.genecards.org searches (Excel sheet in Additional file 1) were manually imported to the database. In order to be processed by the database microarray data have to contain: probe set, accession, description, intensity values, absent/present call and gene name, in a text file. In brief we have used affylmGUI [23] to RMA normalized gene expression data and compared the gene expression of each hematopoetic population with OP9 cells with P-value <0.01. The obtained gene lists was then analyzed in GCINT to extract existing receptor-ligands based on presence call in at least one population. In order to compare the expression of relevant genes in NIH3T3 and MC3T3 cells, the RMA normalized expression values were extracted from dChip [24] and inserted into the tables (Table 1, 2, 3, 4, 5, 6, 7 and 8 and additional files 2, 3, 4, 5, 6, 7, 8 and 9). Gene expression data from hematopoietic cells were collected from [25-28] (GO accession numbers GSE8407, GSE7302 and GSE11110) while gene expression data from OP9 stroma cells, NIH3T3 fibroblasts and MC3T3 Osteoblasts were generated for this study and has been deposited in GO (accession number GSE19729). The Mirosoft Acess database will be freely available upon request.

**Table 1:** Analysis of communication pathways for Long Term Hematopoietic Stem Cells (LT-HSC)

Gene expressed in LT-HSC	Mean Intensity	Corresponding gene in stroma	Mean Intensity	NIH3T3	Osteoblasts
activin receptor IIB	267	bone morphogenetic protein 4	1335	66	252
bone morphogenetic protein 4	169	bone morphogenetic protein receptor, type 1A	497	274	384
chemokine (C-X-C motif) receptor 4	372	chemokine (C-X-C motif) ligand 12	12222	23	798
epidermal growth factor receptor	149	epiregulin	422	288	153
insulin-like growth factor 2	105	insulin-like growth factor 2 receptor	535	1772	1704
Integrin alpha 4	1675	junction adhesion molecule 2	201	16	26
integrin beta 1 (fibronectin receptor beta)	307	junction adhesion molecule 2	201	16	26
integrin beta 2	558	junction adhesion molecule 3	152	42	204
jagged 2	222	Notch gene homolog 3 (Drosophila)	367	161	48
oncostatin M	115	oncostatin M receptor	1151	1349	131
epidermal growth factor receptor	149	transforming growth factor alpha	261	13	21
Integrin alpha 4	1675	vascular cell adhesion molecule 1	692	182	929
integrin beta 1 (fibronectin receptor beta)	307	vascular cell adhesion molecule 1	692	182	929
integrin beta 7	329	vascular cell adhesion molecule 1	692	182	929
kinase insert domain protein receptor	311	vascular endothelial growth factor A	179	1006	169

**Table 2:** Analysis of communication pathways for Multipotent progenitors (MPP)

Gene expressed in MPP	Mean Intensity	Corresponding gene in stroma	Mean Intensity	NIH3T3	Osteoblasts
activin receptor IIB	183	bone morphogenetic protein 4	1335	66	252
Bone morphogenetic protein 15	413	bone morphogenetic protein receptor, type 1A	327	377	287
bone morphogenetic protein 4	112	bone morphogenetic protein receptor, type 1A	327	377	287
chemokine (C-X-C motif) receptor 4	264	chemokine (C-X-C motif) ligand 12	12222	23	798
epidermal growth factor receptor	231	epiregulin	422	288	153
insulin-like growth factor 2	271	insulin-like growth factor 2 receptor	535	1772	1704
interleukin 1 receptor, type I	123	interleukin 1 receptor antagonist	3515	16	10
integrin alpha 4	278	junction adhesion molecule 2	201	16	26
integrin beta 1 (fibronectin receptor beta)	180	junction adhesion molecule 2	201	16	26
integrin beta 2	507	junction adhesion molecule 3	152	42	204
oncostatin M	157	oncostatin M receptor	1151	1349	131
epidermal growth factor receptor	231	transforming growth factor alpha	261	13	21
integrin alpha 4	278	vascular cell adhesion molecule 1	692	182	929
integrin beta 1 (fibronectin receptor beta)	180	vascular cell adhesion molecule 1	692	182	929

**Table 3:** Analysis of communication pathways for Lymphoid primed Multipotent Progenitors (LMPP)

Gene expressed in LMPP	Mean Intensity	Corresponding gene in stroma	Mean Intensity	NIH3T3	Osteoblasts
activin receptor IIA	131	bone morphogenetic protein 4	1335	66	252
activin A receptor, type 1	107	bone morphogenetic protein 4	1335	66	252
bone morphogenetic protein 4	277	bone morphogenetic protein receptor, type 1A	497	274	384
epidermal growth factor receptor	189	epiregulin	422	288	153
insulin-like growth factor 2	139	insulin-like growth factor 2 receptor	535	1772	1704
interleukin 2 receptor, gamma chain	2115	interleukin 7	189	92	255
oncostatin M	178	oncostatin M receptor	1151	1349	131
chemokine (C-X-C motif) receptor 4	290	chemokine (C-X-C motif) ligand 12	12222	23	798
epidermal growth factor receptor	189	transforming growth factor alpha	261	13	21
tumor necrosis factor	102	tumor necrosis factor receptor superfamily, member 1a	1974	959	1143
tumor necrosis factor	102	tumor necrosis factor receptor superfamily, member 1b	108	721	108
integrin alpha 4	268	vascular cell adhesion molecule 1	692	182	929
integrin beta 7	453	vascular cell adhesion molecule 1	692	182	929
integrin beta 1 (fibronectin receptor beta)	173	vascular cell adhesion molecule 1	692	182	929
integrin beta 1 (fibronectin receptor beta)	173	junction adhesion molecule 2	201	16	26
integrin alpha 4	268	junction adhesion molecule 2	201	16	26
integrin beta 2	484	junction adhesion molecule 3	152	42	204

**Table 4:** Analysis of communication pathways for Common Lymphoid Progenitors (CLP)

Gene expressed in CLP	Mean Intensity	Corresponding gene in stroma	Mean Intensity	NIH3T3	Osteoblasts
interleukin 2 receptor, gamma chain	3021	interleukin 7	189	92	255
chemokine (C-X-C motif) receptor 4	2121	chemokine (C-X-C motif) ligand 12	12222	23	798
integrin alpha 4	1982	vascular cell adhesion molecule 1	692	182	929
integrin alpha 4	1982	junction adhesion molecule 2	201	16	26
integrin beta 2	920	junction adhesion molecule 3	152	42	204
Bone morphogenetic protein 15	645	bone morphogenetic protein receptor, type 1A	497	274	384
chemokine (C-C motif) receptor 9	443	chemokine (C-C motif) ligand 25	123	55	100
insulin-like growth factor 2	282	insulin-like growth factor 2 receptor	535	1772	1704
epidermal growth factor receptor	193	epiregulin	422	288	153
epidermal growth factor receptor	193	transforming growth factor alpha	261	13	21
interleukin 1 receptor, type I	180	interleukin 1 receptor antagonist	3515	16	10
activin receptor IIB	166	bone morphogenetic protein 4	1335	66	252
interleukin 1 receptor, type II	127	interleukin 1 receptor antagonist	3515	16	10
oncostatin M	112	oncostatin M receptor	1151	1349	131

**Table 5:** Analysis of communication pathways for Granolocyte Monocyte Progenitors (GMP)

Gene expressed in GMP	Mean Intensity	Corresponding gene in stroma	Mean Intensity	NIH3T3	Osteoblasts
chemokine (C-X-C motif) receptor 4	768	chemokine (C-X-C motif) ligand 12	12222	23	798
epidermal growth factor receptor	182	epiregulin	422	288	153
interleukin 2 receptor, gamma chain	869	interleukin 7	189	92	255
integrin alpha 4	171	junction adhesion molecule 2	201	16	26
integrin beta 1 (fibronectin receptor beta)	122	junction adhesion molecule 2	201	16	26
integrin beta 2	969	junction adhesion molecule 3	152	42	204
epidermal growth factor receptor	182	transforming growth factor alpha	261	13	21
integrin alpha 4	171	vascular cell adhesion molecule 1	692	182	929
integrin beta 1 (fibronectin receptor beta)	122	vascular cell adhesion molecule 1	692	182	929
neuropilin 1	120	vascular endothelial growth factor A	179	1006	169
neuropilin 1	120	vascular endothelial growth factor B	174	191	639

**Table 6:** Analysis of communication pathways for progenitor B-cells (Pro-B)

Gene expressed in ProB	Mean Intensity	Corresponding gene in stroma	Mean Intensity	NIH3T3	Osteoblasts
activin receptor IIA	139	bone morphogenetic protein 4	1335	66	252
activin receptor IIB	178	bone morphogenetic protein 4	1335	66	252
CD28 antigen	439	CD80 antigen	462	38	71
epidermal growth factor receptor	193	Epiregulin	422	288	153
insulin-like growth factor 2	113	insulin-like growth factor 2 receptor	535	1772	1704
interleukin 2 receptor, gamma chain	3348	interleukin 7	189	92	255
L1 cell adhesion molecule	596	neural cell adhesion molecule 1	129	740	545
oncostatin M	143	oncostatin M receptor	1151	1349	131
platelet derived growth factor, B polypeptide	122	platelet derived growth factor receptor, alpha polypeptide	2140	1066	445
platelet derived growth factor, B polypeptide	122	platelet derived growth factor receptor, beta polypeptide	1829	505	1782
chemokine (C-C motif) receptor 2	141	chemokine (C-C motif) ligand 2	239	3362	362
chemokine (C-C motif) receptor 9	376	chemokine (C-C motif) ligand 25	123	55	100
chemokine (C-X-C motif) receptor 4	702	chemokine (C-X-C motif) ligand 12	12222	23	798
epidermal growth factor receptor	193	transforming growth factor alpha	261	13	21
integrin alpha 4	413	vascular cell adhesion molecule 1	692	182	929
integrin beta 7	2879	vascular cell adhesion molecule 1	692	182	929
integrin beta 1 (fibronectin receptor beta)	130	vascular cell adhesion molecule 1	692	182	929
neuropilin 1	333	vascular endothelial growth factor A	275	1872	387
kinase insert domain protein receptor	389	vascular endothelial growth factor A	275	1872	387
integrin beta 1 (fibronectin receptor beta)	130	junction adhesion molecule 2	201	16	26
integrin alpha 4	413	junction adhesion molecule 2	201	16	26
integrin beta 2	4429	junction adhesion molecule 3	152	42	204

**Table 7:** Analysis of communication pathways for pre-B cells (pre-B)

Gene expressed in PreB	Mean Intensity	Corresponding gene in stroma	Mean Intensity	NIH3T3	Osteoblasts
chemokine (C-X-C motif) receptor 4	1526	chemokine (C-X-C motif) ligand 12	12222	23	798
epidermal growth factor receptor	177	Epiregulin	422	288	153
interleukin 2 receptor, gamma chain	2147	interleukin 7	189	92	255
integrin alpha 4	199	junction adhesion molecule 2	201	16	26
integrin beta 2	1438	junction adhesion molecule 3	152	42	204
oncostatin M	101	oncostatin M receptor	1151	1349	131
epidermal growth factor receptor	177	transforming growth factor alpha	261	13	21
integrin alpha 4	199	vascular cell adhesion molecule 1	692	182	929

**Table 8:** Analysis of communication pathways for mature B-cells (B-cells)

Gene expressed in MatureB	Mean Intensity	Corresponding gene in stroma	Mean Intensity	NIH3T3	Osteoblasts
chemokine (C-X-C motif) receptor 4	1275	chemokine (C-X-C motif) ligand 12	12222	23	798
epidermal growth factor receptor	145	Epiregulin	422	288	153
interleukin 2 receptor, gamma chain	2746	interleukin 7	189	92	255
Integrin alpha 4	1640	junction adhesion molecule 2	201	16	26
integrin beta 1 (fibronectin receptor beta)	235	junction adhesion molecule 2	201	16	26
integrin beta 2	1781	junction adhesion molecule 3	152	42	204
lymphotoxin A	540	lymphotoxin B receptor	965	285	519
transforming growth factor, beta 1	718	Similar to Ornithine decarboxylase (ODC)	197	146	14
epidermal growth factor receptor	145	transforming growth factor alpha	261	13	21
transforming growth factor, beta 1	718	transforming growth factor, beta receptor II	171	85	253
transforming growth factor, beta 1	718	transforming growth factor, beta receptor III	3653	3429	1152
lymphotoxin A	540	tumor necrosis factor receptor superfamily, member 1a	1974	959	1143
lymphotoxin A	540	tumor necrosis factor receptor superfamily, member 1b	108	721	108
Integrin alpha 4	1640	vascular cell adhesion molecule 1	692	182	929
integrin beta 1 (fibronectin receptor beta)	235	vascular cell adhesion molecule 1	692	182	929
integrin beta 7	942	vascular cell adhesion molecule 1	692	182	929

Tables 1, 2, 3, 4, 5, 6, 7 and 8. Receptor ligand pairs identified as possible means of communication between a model stroma cell line OP9 and sorted hematopoietic cells from the mouse bone marrow. Expression values observed in fibroblastic NIH3T3 and osteoblastic MC3T3 cells are included to allow for the identification of stromal cell specific interactions. The diagram only display receptor ligand pairs were genes on the hematopoietic array as well as of the stromal cells arrays have a normalized expression value above 100. Full lists of potential interactions can be found in Additional files 2, 3, 4, 5, 6, 7, 8 and 9. For sorted cells as well as OP9 the value is a mean out of 2 experiments for complete list of genes for each cell type see Additional files 2, 3, 4, 5, 6, 7, 8 and 9.

### Tissue culture conditions and cell lines

BAF3 cells were maintained in RPMI (PAA) supplemented with 10% fetal calf sera (FCS) (Hyclone), 10mM HEPES (Gibco), 50 μM 2-mercaptoethanol (Gibco), 50 μg/ml Gentamicin (Invitrogen). The culturing media was also supplemented with 5% conditioned media from confluent WEHI3 cells, as source of interleukin-3. 230-238 cells were grown in RPMI supplemented with 10% FCS (Hyclone) and 50 μg/ml Gentamicin (Invitrogen). FDC-P1 cells were maintained in Iscove's Modified Dulbecco's Media (PAA) supplemented with 10% FCS (Hyclone) 10% WEHI3 conditioned media and 50 μg/ml Gentamicin (Invitrogen). NIH3T3 and MC3T3 cells were propagated in Dulbecco's Modified Eagle Medium (Gibco) supplemented with 10% FCS (Hyclone) and 50 μg/ml Gentamicin (Gibco). OP9 and OP9 Strawberry were maintained in OptiMEM (Gibco) supplemented with 10% FCS, 50 μg/ml Gentamicin (Invitrogen). Adherent cells were treated with 0,1% Trypsin/EDTA (Gibco) before passage. All cells were maintained in 37°C 5% CO_2_.

### Isolation and purification of cells

Purification of LSK cells was performed as described in [28]. Femur and tibia were taken from 10-16 week old C57BL/6 mice, crushed to obtain suspension cells. BM cells were enriched in KIT^+ ^(CD117+) cells using anti-CD117 immuno magnetic beads (Miltenyi Biotechnology) and separated in an Auto MACS column (Miltenyi Biotechnology). BM cells were stained for Sca-1 Fitc (EB-161.7), CD117 APC (2B8) and Linage PeCy5: CD3 (17A2), CD4 (GK1.5), Gr1 (RB6-8C5), CD19 (6D5), Mac1(M1/70), NK.1(PK136) and Ter119 (Ter 119) (All from Biolegend) after blocking unspecific binding by CD 16/32 (93) antibody (Biolegend). Sca^+ ^Kit^+ ^Lin^- ^cells were sorted with BD FACS Aria Special Order System™. Animals were housed in the animal facility at Linköping University and the experiments were conducted according to the instructions and with the permission from Linköping ethical committee.

### Generation of Strawberry fluorescent OP9

mStrawberry vector was kindly provided by Dr R. Tsien UCSD. The mStrawberry gene was cloned between the BamH1 and EcoR1 sites in a pBabe-Puro vector. In order to produce retrovirus, pBabe Strawberry encoding plasmid was transfected in to Phoenix packaging cells using Lipofectin (Invitrogen). Transfected cells were selected in 2 μg/ml puromycin (Sigma Aldrich) for 1 week. Viral supernatant were collected and used for infection of OP9 cells by incubation for 8 hours in the presence of 0,5 ug/ml polybren (Sigma Aldrich). Infected OP9 cells were sorted for high expression of Strawberry detected by FACS Aria Special Order™ System using TxRed channel.

### In vitro B-lymphoyte differentiation experiments

15 000 OP9 or 20 000 NIH3T3 cells were suspended in 1 ml optiMEM (Gibco) supplemented with 10% FCS (Hyclone), 25 mM HEPES (Gibco), 50 μg/ml Gentamicin (Gibco) 50 μM 2-mercaptoethanol (Gibco) and seeded into each well of a 24 well plate. LSK cells were sorted, suspended in optiMEM (Gibco) supplemented with 10% FCS (Hyclone), 25 mM HEPES (Gibco), 50 μg/ml Gentamicin (Gibco) 50 μM 2-mercaptoethanol (Gibco) at a density of 100 cells/ml. This cell suspension was used to replace the medium in the wells containing OP9 or NIH3T3 cells that within 4 hours adhere to the bottom of the wells to form a feeder layer. The cultures were then supplemented with cytokines IL-7, FL, (Both at a concentration of 10 ng/ml) CXCL12, (All from Peprotech) BMP4, IL1ra (All at a concentration of 30 ng/ml) (Both from Prospec) as indicated. Cells were maintained in 37°C 5% CO_2 _for 7 days, where after 50% of the upper layer of media was replaced and 1 ml of new cytokine containing media were added. At day 14 suspension cells were gently removed from the wells to be stained with B220 PE (RA36B2) (BecktonDickinson), CD19 PECy5 (6D5), Mac1 FITC (M/70) (All from Biolegend), analyzed on FACS Calibur (BecktonDickinson). The flow data was further analyzed with the FlowJo (Tree star Incorparated) software.

### Microarray analysis

RNA was prepared using RNeasy mini kit with DNase treatment of the column (Qiagen). Concentration of obtained RNA was measured by Nanodrop™ and the RIN value were estimated on the Bioanalyzer (Agilent™). RNA was labeled and amplified according to Affymetrix™ GeneChip Expression Analysis Technical Manual, and hybridized to GeneChip Mouse Genome 430 2.0 Array. Chips were scanned using Affymetrix GeneChip™ Scanner. The data were analyzed using dChip http://www.dchip.org software.

### Gene Expression analysis using real time quantitative PCR

RNA was extracted from sorted OP9 mStrawberry cells as described above. cDNA was generated by annealing 1 μg of total RNA in1,5 mM random hexamer, 5× first strand buffer,100 mM DTT, 5 mM dNTP, 40U RNAse out, 200U superscript III (All from Invitrogen), in a total volume of 20 μl. Real time qPCR was conducted with TaqMan™ technology (Applied Biosystems), the threshold cycles for the endogenous control was set to 0,05. All experiments were conducted in triplicates and normalized against HPRT expression. 2^ΔΔCT ^was calculated as follows, where G.O.I indicates gene of interest ΔΔC_T _= (C_TG.O.I (control)_- HPRT) - (C_TG.O.I (sample) _- HPRT) Control were cells in parallel culture with conditioned media from co-culture sample. Oligonucleotides for quantitative TaqMan real time PCR; Hprt: Mm 00446968_m1, Spib:Mm 01719550_s1, Nov: Mm 00456855_m1, Cxcl10: Mm 00445235_m1 was ordered from AppliedBiosystems™.

## Results

### Genomics Based Cell Interaction (GCINT) analysis allows for a rapid analysis of potential receptor/ligand involvement in the communication between two cell types

**Figure 1:**
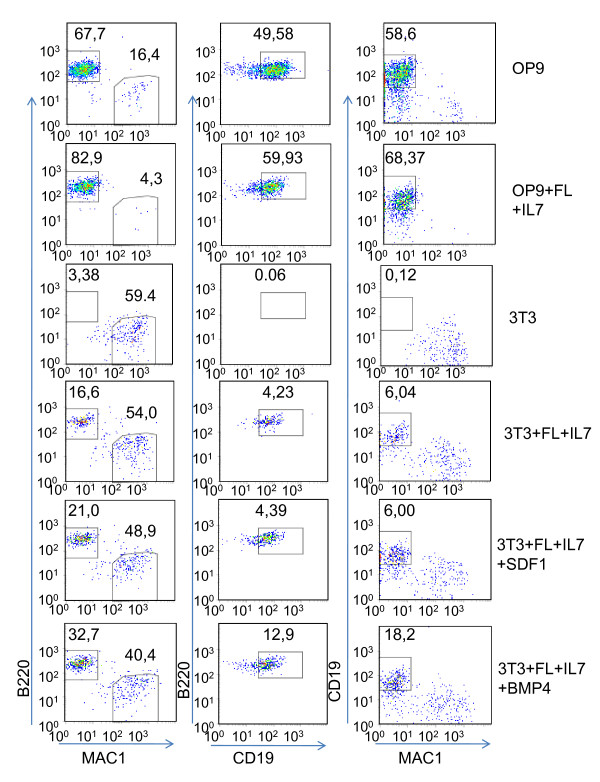
**Differential ability of OP9 and NIH3T3 cells to stimulate B-cell development from hematopoetic progenitor cells**. Representative FACS plots of the cells generated after 14 days of incubation of 100 LSK cells on OP9 or NIH3T3 cells with the indicated addition of cytokines. The cells were analyzed with regard to the expression of CD19, B220, and MAC1 as indicated in the diagrams. The numbers in the panels represents average percentages of different cell subsets obtained under the defined conditions after analysis of at least three independent experiments.

The communication between two cells like the B cell progenitors and the stromal cells is likely to involve a highly complex set of interactions driving proliferation and differentiation of the hematopoietic cells. In order to get a more complete picture over the possible communication pathways during differentiation of B-lineage cells, we created a database with information about 400 known receptor ligand pairs using GeneCards http://www.genecards.org as our major source of information (Excel sheet attached as Additional file 1). The involved gene products were coupled to their locus link number and further to Affymetrix Mouse Genome 430 2.0 Array probe sets. This database was created so to allow for the match of two microarray data sets to unravel potential pathways of communication between the two cell types represented by the array data. GCINT show the gene names, intensity values for the probe sets, locus link accessions and descriptions for the two matched arrays. These values can then be copied to an excel sheet for further analysis. The simplicity of this method allows for investigations of rather large data sets to gain insight to the dynamics of cell-cell communication during biological processes such as development. In order to investigate temporal changes in communication during the path of B-cell development, we sorted and extracted gene expression information by microarray analysis from a variety of hematopoietic progenitor cells in the mouse BM. Long-term hematopoietic stem cells (LT-HSCs) were sorted based on low expression of lineage markers (Lin^-^) (Please see materials and methods for details) and expression of SCA1 and KIT (LSK) but lack of surface expression of CD34 and FLT3 [13,29]. Multipotent progenitors (MPP) were sorted based on a LSKCD34^+^FLT3^- ^phenotype while Lymphoid primed multipotent progenitors (LMPPs) were defined as LSK cells with expression of both CD34 and FLT3 [13,29]. Granulocyte/Monocyte progenitors (GMP) was sorted based on expression of CD34, KIT and FCγ RII/III [30,31] while Common lymphoid progenitors (CLPs) lack the expression of lineage markers, has reduced levels of KIT and SCA as compared to the multipotent progenitors and express IL-7 receptor on the cell surface [32]. The earliest B-lineage restricted progenitors (pro-B) were sorted based on a B220^+ ^CD43^+ ^CD19^+ ^IgM^- ^surface phenotype while pre-B cells were defined as B220^+ ^CD43^- ^CD19^+ ^IgM^-^cells from mouse bone marrow and mature B-cells as CD19^+ ^IgM^+ ^cells from mouse spleen [33]. For all the populations RNA was extracted and subjected to two rounds of linear amplification where after the resulting cRNA was hybridized to a UA430_2 Affymetrix™ oligonucleotidearrays. In order to obtain gene expression information from a relevant mesenchymal cell, we used the same micro-arrays to analyze the gene expression pattern in OP9 stromal cells, with ability to support B-cell development *in vitro*, and NIH3T3 cells, a fibroblast unable to perform this task under our standard conditions (Figure 1). In addition we included gene expression data from the osteoblast cell line MC3T3, representing a third type of BM cell. The output of the GCINT analysis will be highly dependent on the gene lists imported to the program. This creates a large flexibility and opens the possibility to create the gene list used for analysis in a way suitable for the scientific question at hand. In our specific case, we aim to identify factors secreted by stroma cells that may effect the maturation of B-lymphoid cells. Therefore we made the presumption that genes encoding crucial growth factors secreted by stroma cells, would not be expressed by the hematopoetic cells themselves. We then used RMA normalized data from OP9 cells and hematopoetic progenitors and select genes based on differential gene expression (p < 0-01) in the different cell types (Please see Materials and Methods for details). This generated one list of genes for each hematopoietic population. These lists were imported into GCINT and receptor ligand pairs were extracted. This revealed several possibilities for interactions between the different hematopoietic progenitors and the stromal cells (Table 1, 2, 3, 4, 5, 6, 7, 8 and Additional files 2, 3, 4, 5, 6, 7, 8 and 9). Some receptor ligand pairs such as Integrinα 4-VCAM1 were expressed at all stages of hematopoietic development while others were more restricted to defined stages of differentiation. Thus, a genomics based analysis provides information about potential stage specific interactions between hematopoietic progenitors and stromal cells.

### BMP4 and support B-cell development in vitro

**Figure 2:**
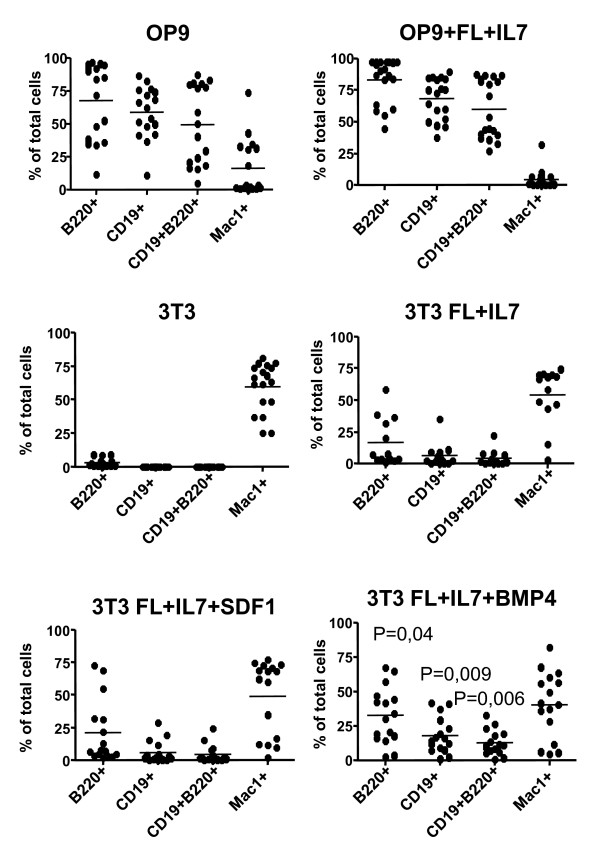
**BNP4 stimulates B-cell development from early bone marrow progenitor cells**. Diagrams displaying the cellular composition of cultures as judged by FACS analysis of B220, CD19 and MAC-1 surface expression obtained by *in vitro *differentiation of 100 LSK cells. The progenitors were incubated for 14 days with either OP9 or NIH3T3 cells and the indicated cytokines. Each dot in the diagrams represents one well and data are collected from at least three independent experiments (n = 13-18). Line indicates the average of all the analyzed wells and the P values has been calculated as compared to the cellular composition obtained on NIH3T3 cells with the addition of FL and IL7.

Knowing that our analysis extracted information about potential stage specific cell interactions, we wanted to investigate if this approach allowed for the identification of B-lineage growth factors. To this end, we develop an experimental system based on two functionally different support cells differing in their ability to support the development of B-lineage cells from immature hematopoietic progenitor cells. Such a model system allow us to compare gene expression patterns in the two cell lines to identify genes associated with an ability to support B-cell development. Further, this opens the possibility to functionally investigate the role for the identified factors by the addition of the potentially involved proteins in co-culture experiments. The stromal cell line OP9 is known to be a powerful stimulator of B-cell development *in vitro *so as a control, we used standard growth conditions for immature LSK cells on OP9 cells with optiMEM supplemented with FCS. Cultivation of LSK cells on OP9 resulted in a high percentage of CD19^+^B220^+ ^cells (Figure 1 (OP9) and Figure 2) and even though the addition of FL and IL7 did not have a major impact of the cellular composition of the cultures (Figure 1 (OP9+FL+IL7)), the cell numbers were increased (Data not shown). In contrast, without the addition of any cytokines, more than 50% of the hematopoietic cells generated on NIH3T3 cells expressed MAC1 (Figure 1 (3T3)) indicating that they had developed into myeloid lineage cells. A few B220^+ ^but no CD19^+ ^cells, representing the committed progenitors [33], were detected. This suggests that NIH3T3 cells are unable to support the development of CD19^+ ^B-lymphoid cells from multipotent hematopoietic progenitors. The two cytokines FLT3 and IL7 has been shown able to allow for B-lineage development from LSK cells *in vitro *[34] and the inclusion of these growth factors into the NIH3T3/LSK co-cultures resulted in an increased percentage of B220^+ ^cells and also generation of CD19^+ ^cells (Figure 1 (3T3+FL+IL7)). These experiments suggested that OP9 cells but not NIH3T3 cells express factors that are involved in the generation of B-lymphoid cells from multipotent progenitor cells. We next used our receptor ligand analysis in order to identify genes encoding growth factors that were differentially expressed in OP9 and NIH3T3 cells and were mRNA coding for a receptor was expressed in early progenitors and B-lineage cells. One factor exclusively expressed in OP9 cells, was CXCL12 presumably interacting with the CXCR4 receptor on the B-lineage cells (Table 6 and 7). This factor has been suggested to stimulate B cell development and growth [6,7], however, in our experimental setting, this factors was unable to support development of CD19^+ ^cells from multipotent progenitors to any significantly higher level than FL and IL-7 (Figure 1 (3T3+FL+IL7+CXCL12) and Figure 2). A second cytokine, BMP4, with a possibility to interact with the BMPR on the pro-B cells, was expressed to a higher level in the OP9 cells as compared to NIH3T3 cells. In contrast to CXCL12, the addition of BMP4 resulted in an increased formation of both CD19^+ ^and CD19^+ ^B220^+ ^cells as compared to the FL and IL7 stimulated control cultures (p < 0,001)(Figure 1 (NIH3T3+FL+IL7+BMP4) and Figure 2). Thus, this approach allows for the identification of novel growth factors involved in the development of B-lymphoid cells.

### Contact with hematopoietic cells modulates gene expression patterns in the stromal cells

**Figure 3:**
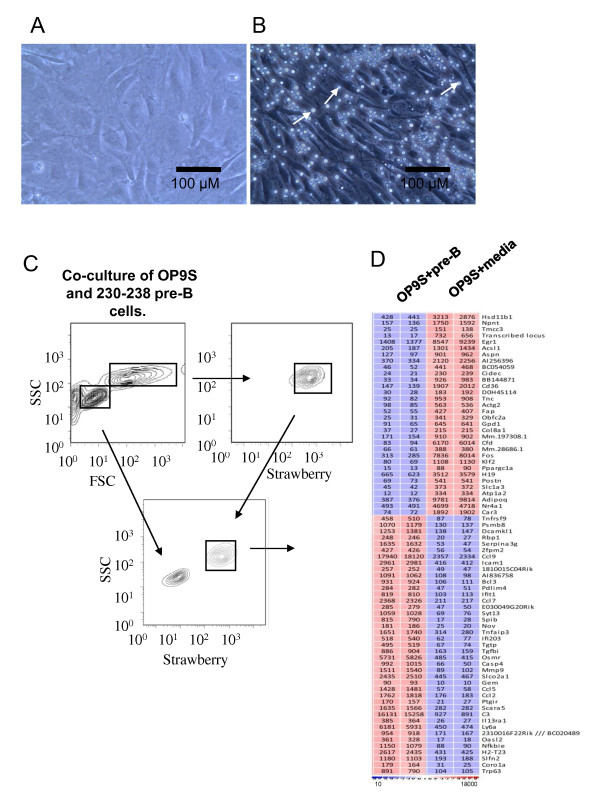
**B-cell progenitors modulate the behavior of OP9 stromal cells *in vitro***. Panel (A-B) display pictures of OP-9 cells grown in parallel cultures with either conditioned media (A) or in direct contact with pro-B cells (B) generated by *in vitro *differentiation of LSK cells. The pictures are taken from the same six well tissue culture plates with 200 times amplification and under the same light conditions (Leica DFC290). The black bar indicates 100 μM. Panel (C) shows a representative sorting experiment where trypsin/EDTA treated co-cultures of OP9S and pro-B cells are separated based on forward scatter and Strawberry expression. Panel (D) display a cluster analysis of RMA normalized gene expression patterns in OP9S cells incubated with either conditioned media or the pre-B cell line 230-238. The analysis displays genes with an expression level above 100 and with at least 5-fold difference in expression in the two experiments with P-value < 0.05. Red indicates high and blue low expression.

**Figure 4:**
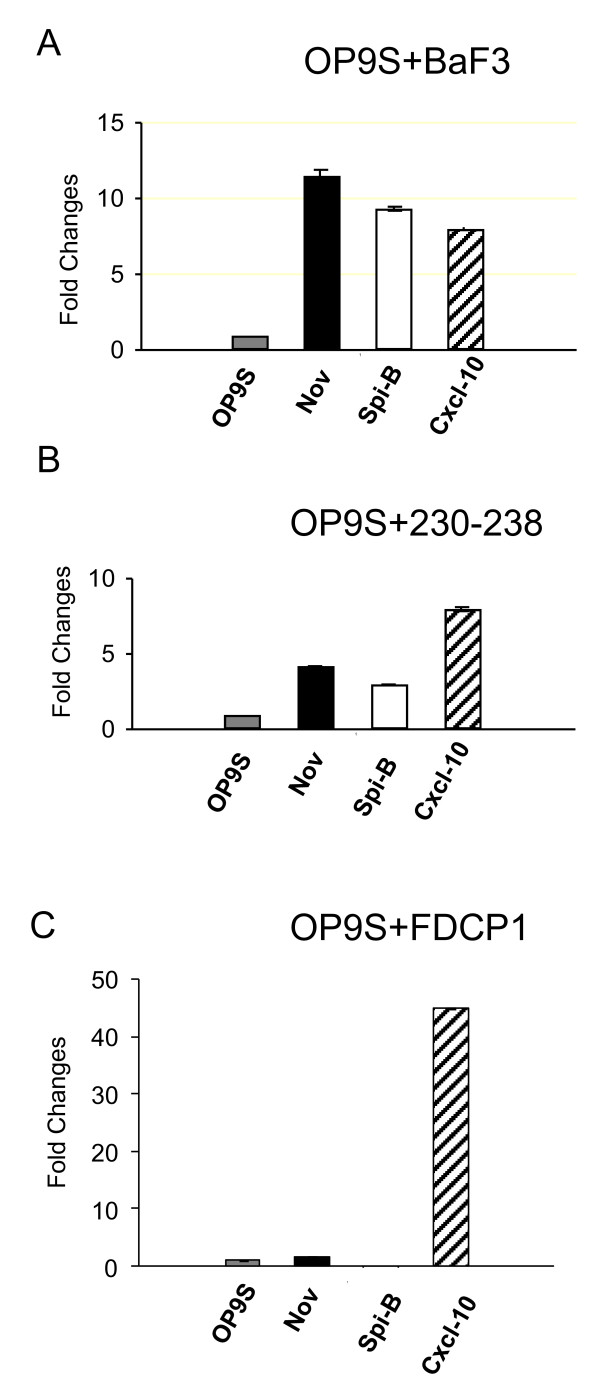
**Co-culture of cell lines representing different hematopoietic lineages induces differential gene expression patterns in OP9 cells**. The diagrams display HPRT normalized Q-PCR data from OP9S cells generated by co-incubation of hematopoietic cell lines or filtered media from the parallel co-culture experiments (OP9S). The expression pattern in the control cells were set to one for each of the *Nov *(Black bar), *SpiB *(White bar) or *Cxcl10 *(Striped bars) and the relative induction/reduction is indicated. Error bars indicate experimental variation in one out of two or three experiments.

Another possibility created by this type of data analysis is to investigate any apparent directionality of communication and even though the analysis of the pro-B/OP9 interaction suggests that the majority of the information flow is from the stroma to the hematopoietic cell, there exist several possibilities for communication in the opposite direction. In order to investigate if hematopoietic cells would be able to induce a cellular response in stromal cells, we incubated either *in vitro *expanded primary B-cell progenitors or a pre-B cell line with OP9 stromal cells for 7 days. This revealed that co-incubation with either transformed B-cell lines (Data not shown) or primary Pro-B cells generated by *in vitro *differentiation of LSK cells, resulted in a change of morphology of the stromal cells (Figure 3A,B). This effect was not detected when OP9 cells were incubated with conditioned media from a parallel co-culture experiment suggesting that this was not due to alterations in nutrient concentrations in the media or due to secreted factors but a result of cell-cell contact or proximity. The control culture contained big, rounded cells while the OP9s incubated with B-lineage progenitors had adapted a different morphology with elongated cells (Figure 3A,B). These data suggest that the B-cell progenitor cells were able to modulate the morphological characteristics of the OP9 stromal cells. In order to investigate this further, we wanted to extract RNA from the stromal cells in the co-culture and the corresponding control culture. This experiment was complicated by that the B-cell progenitors attached firmly to the OP9 cells demanding trypsin/EDTA treatment of the cells in order to generate the homogenous single cell suspension needed to separate the OP9 cells from the hematopoietic cells. Treament with trypsin/ETDA reduces the possibility to sort cells by Flow Cytometry based on cell surface marker expression. Hence, in order to be able to purify the OP9 cells for molecular analysis, we did a retroviral infection of OP9 cells with the red fluorescent protein - mStrawberry [35]) (OP strawberry, (OP9S)). This allowed us to sort out the red stromal cells (Figure 3C) and separate them from the hematopoietic cells in order to investigate potential changes in gene expression patterns in the OP9 cells. To this end, we incubated OP9S cells with 230-238 pre-B cells for 7 days and the gene expression pattern was compared to that of cells obtained by parallel incubation with the conditioned media by microarray analysis (Figure 3D). This suggested that several genes in the stromal cells were induced by the direct contact with the B-cell progenitor cell line while another set of genes were expressed at a lower level in the co-cultured OP9 cells. Among the genes induced by the pre-B cells were several chemokine genes including *Ccl-2, -5, -9 *as well as the hematopoietic growth factor Nephrobastoma overexpressed protein (NOV) [36]. We could also detect differential expression of a number of transcription factors including the ets transcription factor SpiB. In order to verify the induction of some genes and to investigate potential differences in the response dependent of the hematopoietic cell type used for co-culture, we performed Q-PCR from sorted OP9S cells incubated for 7 days with pre-pro B cells (BaF3), pre-B cells (230-238) or the myeloid progenitor cell line FDC-P1. Incubation with BaF3 cells resulted in up-regulation of *Nov*, *Spi-B *as well as *Cxcl10 *message (Figure 4A) and even if the level of induction was varying between different experiments (Data not shown), these changes were consistently detected. The same induction pattern was observed using 230-238 pre-B cells (Figure 4B) while the response to the myeloid progenitor cell line FDC-P1 cells differed in that even though a dramatic induction in *Cxcl10 *mRNA could be detected, the expression of *SpiB *or *Nov *message was not increased (Figure 4C). This indicates that the response of the OP9S cells may vary dependent on the cell type used for the co-culture experiment suggesting that different hematopoietic cells may induce specific gene expression patterns in stroma cells.

## Discussion and Conclusions

We here report of how gene expression data can be used to identify molecules involved in cellular synapses. This type of analysis is most helpful for the understanding of complex systems in which cell-cell contact is crucial for the function or development of cells. Our analysis using data from a model stromal cell line and primary hematopoietic cell populations representing different lineages and developmental stages, suggests that even though several communication pathways are shared, we were able to identify potential stage and lineage restricted pathways that may be involved in events such as lineage restriction. Some of these involve known factors such as the IL-7 and CXCL12 pathways, both reported to be important for B cell development [7,15,16,37-39] but the approach taken also allows for the identification of novel factors of potential interest. The methodology opens for a possibility to identify cytokines or surface proteins involved in the regulation of hematopoiesis through comparative analysis of gene expression patterns in functionally different support cells. In this paper we report of such an analysis using fibroblastic NIH3T3 cells, unable to support B-lymphoid development from multipotent progenitor cells, and OP9 cells that are supportive of this process. This allowed us to identify two factors expressed by OP9 but not NIH3T3 cells. Among these has CXCL12 previously been reported to stimulate B cell growth and development [6,7], however, in our co-culture system, the second candidate, BMP4, were the more potent stimulator of B-cell development (Figure 2). A role for BMP4 in hematopoiesis was has been suggested from studies in xenopus embryos as this factor is important for the specification of the first hematopoietic progenitors [40,41]. BMP4 has also been shown to stimulate embryonal stem cells from both mouse [42] and humans [43] to undergo development into hematopoietic lineages. BMP proteins have also been shown to stimulate the growth of primitive human hematopoietic stem cells [44] arguing for a functional role on stem cell expansion. Our data suggest that BMP4 also possess the ability to stimulate development of adult progenitors into B-lymphoid cells. We have not investigated the mechanisms involved in effects of BMP4 or selective abilities of the investigated cytokines to support development and expansion of specific cell types but our findings suggest that our genomics approach reveals relevant functional interactions.

The normal BM stroma is composed of a large variety of cell types with potentially highly specific functions in the regulation of hematpoiesis. In these initial studies we have used a model stromal cell line, OP9, with a high potential to stimulate B-lymphocyte development. This has allowed us to obtain ideas about signaling pathways in the bone marrow, however, in order to fully understand the signaling processes we would need to investigate relevant primary stroma cells from mouse BM. The BM is thought to be organized into anatomical niches harboring blood cells of specific developmental stages and possibly lineages. The most immature cells has been suggested to reside in the endosteum where osteoblasts presumably are involved in the regulation and maintenance of the stem cell pool while more mature cells reside closer to the central region of the bone marrow cavity [4,45-47]. In the case of B-cell progenitors, it has been suggested that they reside in close contact with adventitial reticular cells near to the BM capillaries [8]. Interestingly, it does not appear as if the same stromal cells produce both CXCL12 and IL-7 but that these cytokoines rather are produced by different cells [8]. Thus, in order for the B-cell progenitor to obtain the proper stage specific signals there would exist need for a movement of the developing cells in the BM. This could well be one of the driving forces of cell maturation and niche organization, however, our data suggesting that the stromal cells respond to signals from the hematopoietic cells opens for an alternative or possibly complementary explanation where the cells that in direct contact with the stromal cell stimulates activation of a certain cytokine profile. As the cell mature, the signals from the developing cell changes resulting in new instructions to the stromal cells creating a dynamic system where the progenitors and the stroma directly communicates in order to drive the development of a hematopoietic cell as well as the stroma. In this scenario there would exist plasticity within a certain niche to ensure that all progenitors that enter the compartment are driven to differentiation. Such mechanism could operate on a single cell level, however, since any given stromal cell is likely to be in contact with several progenitor cells at a given time, this would require a synchronized differentiation process and an ability of the stromal cell to return to a ground stage when the hematopoietic cells have left to enter another developmental niche. However, there exist a large need to investigate the plasticity of primary stromal cells or potentially mesenchymal stem cells (MSCs) from the BM. MSCs are multipotent cells capable to differentiate along several pathways to produce for instance adipocytes, chondrocytes and osteoblasts *in vitro *[48]. The different cell fates can be regulated through modifications of tissue culture conditions and it has been reported that HSCs have an ability to induce osteoblast differentiation of multipotent mesenchymal stem cells, thus contributing to the creation of the stem cell niche [49].

Even if the information generated by genomics based cell interaction analysis represents an approximation, we believe that we have been able to show that this approach allows for the generation of biologically relevant and useful information. The development of protocols that allows for the purification of specific stromal cell populations directly from the bone marrow will allow us to feed even more relevant information into the program and thereby increase our possibilities to identify novel stage and lineage specific cell-cell communication pathways *in vivo*.

## Authors' contributions

JZ, AL, SZ, MS and NP has designed the database and extracted information about receptor ligand pairs. JZ, SZ, MS and DB have generated microarray data. JZ, HQ, RM, FH has conducted and analysed in vitro differentiation experiments and JZ has performed Q-PCR analysis and stroma culture experiments. All authors has contributed to the writing of the manuscript. All authors have read and approved the final manuscript.

## Supplementary Material

Additional file 1 **Data base information**. Excel sheet with information about all receptor ligand pairs investigated in this report. Click here for file

Additional file 2 **Receptor ligand interactions between stroma cells and hematopoetic stem cells**. A table with the full information about receptor ligand pairs that may be involved in the communication between stroma and hematopoetic stem cells (HSC). Click here for file

Additional file 3 **Receptor ligand interactions between stroma cells and multipotent progenitor cells**. A table with the full information about receptor ligand pairs that may be involved in the communication between stroma and multipotent progenitors (MPP). Click here for file

Additional file 4 **Receptor ligand interactions between stroma cells and lymphoid primed multipotent progenitors**. A table with the full information about receptor ligand pairs that may be involved in the communication between stroma and lymphoid primed multipotent progenitors (LMPP). Click here for file

Additional file 5 **Receptor ligand interactions between stroma cells and common lymphoid progenitors**. A table with the full information about receptor ligand pairs that may be involved in the communication between stroma and common lymhpoid progenitors (CLP). Click here for file

Additional file 6 **Receptor ligand interactions between stroma cells and granulocyte monocyte progenitors**. A table with the full information about receptor ligand pairs that may be involved in the communication between stroma and granulocyte monocyte progenitors (GMP). Click here for file

Additional file 7 **Receptor ligand interactions between stroma cells and pro-B cells**. A table with the full information about receptor ligand pairs that may be involved in the communication between stroma and pro-B cells. Click here for file

Additional file 8 **Receptor ligand interactions between stroma cells and pre-B cells**. A table with the full information about receptor ligand pairs that may be involved in the communication between stroma and pre-B cells. Click here for file

Additional file 9 **Receptor ligand interactions between stroma cells and B cells**. A table with the full information about receptor ligand pairs that may be involved in the communication between stroma and B cells. Click here for file
